# Molecular characterization of *Brucella* spp. from seropositive herds of cattle farmed at the wildlife–livestock–human interface in Rwanda

**DOI:** 10.3389/fvets.2022.1017851

**Published:** 2022-10-11

**Authors:** Jean Bosco Ntivuguruzwa, Francis Babaman Kolo, Richard Gashururu, Evodie Uwibambe, Vestine Musanayire, Angelique Ingabire, Lydia Umurerwa, Emil Ivan Mwikarago, Henriette van Heerden

**Affiliations:** ^1^Bovine Tuberculosis and Brucellosis Programme, Department of Veterinary Tropical Diseases, Faculty of Veterinary Science, University of Pretoria, Pretoria, South Africa; ^2^Department of Veterinary Medicine, School of Veterinary Medicine, University of Rwanda, Kigali, Rwanda; ^3^Department of Animal Resources and Veterinary Services, Rwanda Agriculture and Animal Resources Board, Kigali, Rwanda; ^4^National Reference Laboratory Division, Department of Biomedical Services, Rwanda Biomedical Centre, Kigali, Rwanda

**Keywords:** *Brucella* spp., dairy cattle, multiplex PCR assays, wildlife–livestock–human interface, Rwanda, seropositive herds

## Abstract

Seroprevalence studies showed that brucellosis is prevalent in cattle in Rwanda with no recent study on the characterization of *Brucella* spp. Therefore, this study aimed to characterize *Brucella* spp. in seropositive herds of cattle farmed at the wildlife–livestock–human interface. Whole blood samples (*n* = 118), milk (*n* = 41), and vaginal swabs (*n* = 51) were collected from 64 seropositive herds. All samples (*n* = 210) were inoculated onto modified Centro de Investigacion y Tecnologia Agroalimentaria (CITA) selective medium. Cultures were analyzed to detect *Brucella* spp. using 16S−23S ribosomal DNA interspacer region (ITS) PCR, the *Brucella* cultures were speciated using AMOS and Bruce-ladder PCR assays. *Brucella* spp. were detected in 16.7% (35/210) of the samples established from the samples using ITS-PCR. The AMOS PCR assay identified mixed *Brucella abortus* and *B. melitensis* (*n* = 6), *B. abortus* (*n* = 7), and *B. melitensis* (*n* = 1) from cultures from blood samples; mixed *B. abortus* and *B. melitensis* (*n* = 1) and *B. abortus* (*n* = 4) from cultures from milk samples; mixed *B. abortus* and *B. melitensis* (*n* = 6), *B. abortus* (*n* = 8), and *B. melitensis* (*n* = 1) from cultures from vaginal swabs. Bruce-ladder PCR assay confirmed *B. abortus* and *B. melitensis* cultures. The isolation of *Brucella* spp. was significantly associated with districts, with the Nyagatare district having more isolates than other districts (*p* = 0.01). This study identified single or mixed *B. abortus and B. melitensis* infections in cattle samples in Rwanda, which emphasizes the need to improve brucellosis control at the wildlife–livestock–human interface and raise the awareness of cattle keepers, abattoir workers, laboratory personnel, and consumers of cattle products.

## Introduction

Brucellosis is a widespread contagious bacterial disease in livestock, wildlife, marine animals, and humans (1). Brucellosis is caused by bacteria of the genus *Brucella* belonging to the family of alpha-2-Proteobacteriaceae (2, 3). The genus was initially subdivided into six classical species based on their intracellular colonization and host species preference (2). The six classical species include *B. melitensis* which affects primarily goats, *Brucella abortus* affecting cattle, *Brucella ovis* affecting sheep, *B. suis* affecting swine and rats, *B. canis* affecting dogs, and *B. neotomae* affecting wood rats (2, 4). Three classical *Brucella* species which are subdivided into biovars (bv.) include *B. abortus* with bv. 1, 2, 3, 4, 5, 6, and 9, *B. melitensis* with bv. 1, 2, and 3, and *B. suis* with bv. 1, 2, 3, 4, and 5 (1).

*Brucella* spp. are 96% genetically homologous (5) but can be distinguished based on their genetic polymorphisms (6–10). Two molecular markers (*omp2a* and *omp2b*) discovered within the outer membrane protein (*omp25*) were used in combination with restriction enzymes to differentiate *Brucella* spp. and some of their biovars (7, 11). Other *Brucella* spp. specific DNA sequences include repetitive extragenic palindromic (REP) (10), two repeated palindromic DNA sequences (BRU: RS1, Bru: RS2) (12), and the insertion sequence (IS) 711 (8). Insertion sequences are mobile genetic elements that code for proteins responsible for their transposition (13). The IS711 that was first discovered in 1993 from *B. ovis* (14) has 35 copies of the element and is different from that of *B. abortus* which has at least 6 copies (9). The IS711 is a unique sequence of *Brucella* spp. with multiple copies of which some occur at species and biovars-specific sites within the chromosomal locus, and this element is the basis of differentiation between *B*. *abortus* (bv. 1, 2, and 4), *B*. *melitensis* (bv. 1, 2, and 3), *B*. *ovis*, and *B*. *suis* bv.1 (AMOS PCR) (15). Furthermore, IS711 is the basis of discrimination between terrestrial *Brucella* spp. and biovars and vaccine strains using Bruce-ladder PCR assay (16, 17). These molecular PCR assays have reduced the long procedure of conventional phenotypic characterization of *Brucella* spp. in developed countries. However, serological methods are still prevailing in most developing countries with a lack of appropriate knowledge, and biosafety facilities (18, 19).

In Rwanda, the control of brucellosis falls under the animal health law which consists of regulations and procedures for reporting infectious diseases, guidelines for animal movement, and the prohibition of illegal slaughtering (20). Apart from this animal health law, there is no other published documentation about the brucellosis control scheme. However, routine serological testing of cattle and small ruminants is performed before important national ruminants trade for distribution to poor families by the government and other non-government organizations (NGOs), and during annual surveillance (once per year) in areas with high dairy production. Vaccination against brucellosis consists of administering RB51 to calves on demand by herders upon payment of US$ 0.5 per dose. Thus, vaccination is not systematic and coordinated at the national level. In Rwanda, the individual seroprevalence of brucellosis in cattle varies from 2.3 to 18.4% (21, 22), and ranged from 6.1 to 25.0% in women with a history of abortions (23, 24). However, apart from the study that characterized *B. abortus* bv. 3 from cattle in 1984 (25), there are no studies on *Brucella* spp. circulating in Rwandan cattle at the wildlife–livestock–human interface. The objective of this study was therefore to characterize the *Brucella* spp. that are circulating in Rwandan cattle farmed at the wildlife–livestock–human interface to document the updated control scheme for brucellosis in Rwanda.

## Materials and methods

### Description of the study area

**Figure 1:**
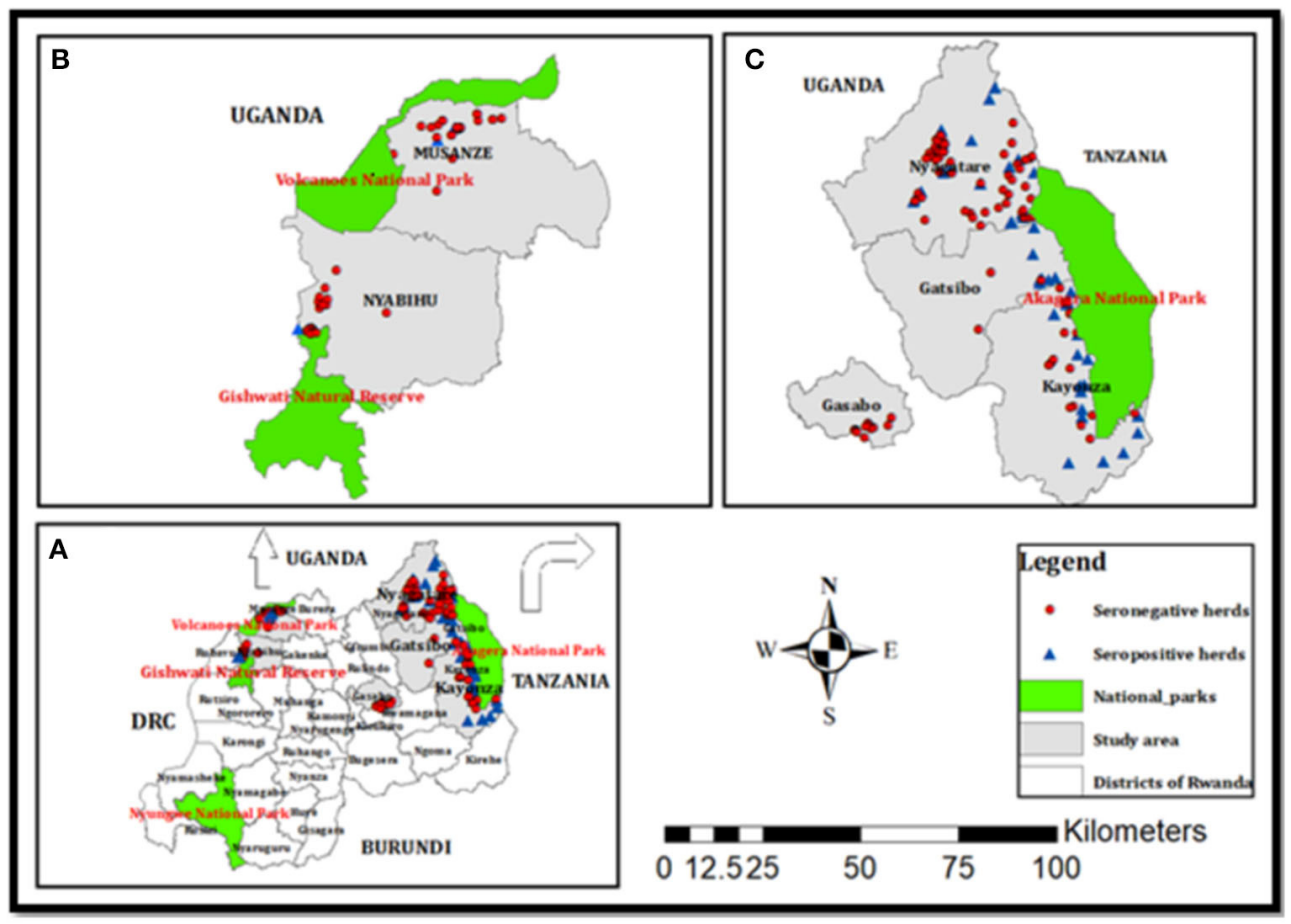
Maps of **(A)** Rwanda with different districts, **(B)** the Musanze and Nyabihu districts border the Virunga and Gishwati national parks, respectively, and **(C)** the Nyagatare, Gatsibo, and Kayonza districts border Akagera National Park, and Gasabo is an urban district with peri-urban areas. Red circles and blue triangles indicate seronegative and seropositive herds found in this study (21).

The study area was selected at the wildlife–livestock–human interface in Rwanda. The interface comprised six districts including Gatsibo, Kayonza, and Nyagatare in the Eastern Province, Musanze in the Northern Province, Nyabihu in the Western Province, and a peri-urban district “Gasabo” of Kigali city. All the districts of Eastern Province border Akagera National Park, which is home to various wildlife animals. Musanze district borders Virunga National Park which accommodates buffaloes and primates, while Nyabihu hosts Gishwati-Mukura National Park which is home to primates and birds (Figure 1). The climate is warmer and drier in the Eastern Province, with annual average rainfalls ranging between 700 and 950 mm, and annual average temperatures ranging between 20 and 21°C. The vegetation is grassland with low inclined hills with an average altitude of 1513.5 m. The climate in the Northern and Western Provinces is the coolest and wettest. The annual rainfall ranges from 1,400 to 1,600 mm with annual average temperatures ranging from 15 to 17°C. The topology in the Northern Province is mountainous with the presence of volcanoes, and the average altitude ranges between 2,000 and 3,000 m (21).

### Study design and sample size

The target population was all cattle, which were brucellosis seropositive (RBT and i-ELISA) plus a few randomly selected cattle with seronegative status but belonging to brucellosis seropositive herds (Figure 1). In the cross-sectional brucellosis seroprevalence study that was previously described (21), the target population was all dairy herds present in the vicinity of national parks and those in the peri-urban areas of Kigali city (Figure 1). During the household visit, for each selected cow, we collected blood in clot activating vacutainers (seroprevalence) and milk from lactating cows or vaginal swabs from non-lactating cows. Due to logistic challenges encountered during sampling, no milk or vaginal swab was collected in the Kayonza district but instead whole blood in 4 ml heparin vacutainer tubes was collected from cattle from the Kayonza district and a few cattle in the Gatsibo district (Table 1). For this bacteriological study, 64 seropositive herds with seropositive cattle (*n* = 183) and seronegative cattle (*n* = 27) were recorded and the samples (*n* = 210) including milk (*n* = 41), vaginal swabs (*n* = 51), and whole blood (*n* = 118) were subjected to bacteriological culture (Table 1). Comparison between the types of samples was not analyzed in this study.

**Table 1:** Types of cultured samples, their brucellosis seropositivity, and their districts of origin.

**Districts**	**Types of samples**			**Seropositivity of cultured samples**		**Total**
	**Milk**	**Vaginal swabs**	**Whole blood**	**Seropositive**	**Seronegative**	
Gasabo	0	0	0	0	0	0
Gatsibo	12	5	33	46	4	50
Kayonza	0	0	84	84	0	84
Musanze	6	7	0	8	5	13
Nyabihu	6	6	0	5	7	12
Nyagatare	17	33	1	40	11	51
Total	41	51	118	183	27	210

### Collection of whole blood, milk, and vaginal swabs

Animals were treated with humane care respecting their welfare. Whole blood was collected aseptically into a 4 ml vacutainer heparin tube from the jugular or tail veins of each animal. At least 20 ml of milk (5 ml from each teat) per cow was collected into the Falcon^®^ 50 ml sterile conical centrifuge tubes (Thermo Fischer Scientific, Johannesburg, South Africa). For non-lactating cattle, a sterile transport swab (Aptaca, Canelli, Italy) was streaked on the walls of the vagina. Each sample was labeled with the corresponding animal identification and transported chilled to the nearest campus of the University of Rwanda. Milk was kept in –20°C while blood and vaginal swabs were kept in the fridge and cultured the following week. Culturing of samples and DNA extraction were done in the biosafety level 3 at National Reference Laboratory, Kigali Rwanda.

### Isolation of *Brucella* spp. from whole blood, milk, and vaginal swabs

The pellet and supernatant of centrifuged milk (3,000 × g at 4°C for 15 min), whole blood, and vaginal swabs were inoculated onto modified CITA plates and incubated at 37°C with a 10% CO_2_ atmosphere as previously described (27). Plates were checked for bacterial growth every day for 3 weeks. For any suspect of *Brucella* spp. based on the morphology of colonies, streaking was performed to have pure colonies from which the DNA was extracted and screened for the presence of a 214 bp interspacer sequence (ITS) of the genus *Brucella* spp.

### DNA extraction and identification of *Brucella* spp. using 16S−23S ribosomal DNA interspacer region (ITS) PCR assay

DNA was analyzed by PCR assays at Rwanda Agriculture Board, Department of Veterinary Services and visualization of PCR products at the Rwanda Biomedical Center, Entomology Laboratory. Genomic DNA was extracted from suspected cultures using the ReliaPrep gDNA tissue Miniprep system following the manufacturer's guidelines (Promega, Madison, USA). The identification of the genus *Brucella* was performed by amplification of the genomic DNA extracted from bacterial colonies using gene-specific primers (Table 2) and a protocol previously developed (28) with slight modifications. *Brucella abortus* strain 544 served as a positive control. The 15 μl PCR reaction mixture contained 1x of MyTaq^TM^ Red PCR Mix (Bioline, South Africa), primers at 0.2 μM, and 2 μl of template DNA (Table 2). The PCR cycling condition was initial denaturation at 95°C for 3 min followed by 35 cycles of denaturation at 95°C for 1 min, annealing at 60°C for 2 min, extension at 72°C for 2 min, and a final extension step at 72°C for 5 min. The primers amplified a 214 bp fragment that was analyzed by electrophoresis using a 2% agarose gel stained with SYBR-safe DNA staining gel (Thermo Fischer, Johannesburg, South Africa) and visualized under UV light.

**Table 2:** Sequences of oligonucleotide primers used for the identification of the genus *Brucella*, the distinction of *Brucella* spp., and differentiation of terrestrial *Brucella* and vaccine strains using 16S−23S ribosomal DNA interspace region (ITS), AMOS, and Bruce-ladder PCR assays.

**PCR name**	**Primer name**	**Sequence (5^′^-3^′^)**	**Targets**	**Size (bp)**	**Conc. (μM)**	**References**
ITS	ITS66f	ACATAGATCGCAGGCCAGTCA	*16s−23s rRNA*	214	0.2	(28)
	ITS279r	ACATAGATCGCAGGCCAGTCA				
A	*B. abortus*	GAC GAA CGG AAT TTT TCC AAT CCC	*IS711*	498	0.1	(15)
M	*B. melitensis*	AAA TCG CGT CCT TGC TGG TCT GA		731	0.1	
O	*B. ovis*	CGG GTT CTG GCA CCA TCG TCG GG		976	0.1	
S	*B. suis*	GCG CGG TTT TCT GAA GGT GGT TCA		285	0.1	
	*IS 711*	TGC CGA TCA CTT AAG GGC CTT CAT		–	0.2	
Bruce-ladder	BMEI0998f	ATC CTA TTG CCC CGA TAA GG	*wboA*	1,682	6.25	(29, 30)
	BMEI0997r	GCT TCG CAT TTT CAC TGT AGC				
	BMEI0535f	GCG CAT TCT TCG GTT ATG AA	*bp26*	450	6.25	(6)
	BMEI0536r	CGC AGG CGA AAA CAG CTA TAA				
	BMEII0843f	TTT ACA CAG GCA ATC CAG CA	*omp31*	1,071	6.25	(31)
	BMEII0844r	GCG TCC AGT TGT TGT TGA TG				
	BMEI1436f	ACG CAG ACG ACC TTC GGT AT	*Deacetylase*	794	6.25	(32)
	BMEI1435r	TTT ATC CAT CGC CCT GTC AC				
	BMEII0428f	GCC GCT ATT ATG TGG ACT GG	*eryC*	587	6.25	(33)
	BMEII0428r	AAT GAC TTC ACG GTC GTTCG				
	BR0953f	GGA ACA CTA CGC CAC CTT GT	*ABC transporter*	272	6.25	(34)
	BR0953r	GAT GGA GCA AAC GCT GAA G				
	BMEI0752f	CAG GCA AAC CCT CAG AAG C	*rpsL*	218	6.25	(35)
	BMEI0752r	GAT GTG GTA ACG CAC ACC AA				
	BMEII0987f	CGC AGA CAG TGA CCA TCA AA	*CRP regulator*	152	6.25	(32)
	BMEII0987r	GTA TTC AGC CCC CGT TAC CT				

### Identification of *Brucella* spp. using AMOS and Bruce-ladder PCR assays

*Brucella abortus, B. melitensis, B. ovis*, and *B. suis* were identified and differentiated using a multiplex AMOS PCR assay as previously described (15). A 25 μl reaction mixture contained 1x MyRaq Red PCR Mix (Bioline, South Africa), four species-specific forward primers, and reverse primer IS711 (Table 2) at a final concentration of 0.1 and 0.5 μM, respectively, and 2 μl of template DNA. Thermocycling conditions included initial denaturation at 95°C for 3 min followed by 35 cycles of denaturation at 95°C for 1 min, annealing at 60°C for 2 min, an initial extension at 72°C for 2 min, and a final extension at 72°C for 5 min. PCR products were analyzed by gel electrophoresis using 2% agarose stained with SYBR safe DNA staining gel (Thermo Fischer, Johannesburg, South Africa) and visualized under UV light.

Vaccine strains and field isolates of *Brucella* spp. were identified and differentiated by a multiplex Bruce-ladder PCR assay developed as previously described (16, 17). A 25 μl PCR reaction contained 1x MyTaq^TM^ Red Mix (Bioline, South Africa), eight species-specific forward and reverse primers at a final concentration of 6.25 μM (Table 2), and 2 μl of template DNA. The PCR cycling conditions included an initial denaturation at 95°C for 3 min followed by 25 cycles at 95°C for 30 s, at 64°C for 45 s, and at 72°C for 3 min, and a final extension step at 72°C for 10 min. PCR products were analyzed by gel electrophoresis using a 2% agarose stained with SYBR safe DNA staining gel (Thermo Fischer, Johannesburg, South Africa) and viewed under UV light.

### Data analysis

Descriptive data were recorded and analyzed in excel spreadsheets. The districts of origin of samples were tested for significant associations with the culture prevalence confirmed by molecular detection of *Brucella* spp. using univariate logistic regression in the EpiInfo software version 7.2.4.0 at the significance level of 95% and *p*-value of 0.05.

### Ethical considerations

This study was approved by the research screening and ethical clearance committee of the College of Agriculture, Animal Sciences and Veterinary Medicine, University of Rwanda (Ref:026/DRIPGS/2017). Ethical clearance was also obtained from the institutional review board of the College of Medicine and Health Sciences, University of Rwanda (N° 006/CMHS IRB/2018). Ethical clearance was also obtained from the Animal Ethics Committee of the Faculty of Veterinary Science, University of Pretoria, South Africa (V004/2018). Informed verbal consents were obtained from district officials and a consent form was signed by each participant before the commencement of this study.

## Results

### Identification of *Brucella* spp. using 16S−23S ribosomal DNA interspacer region (ITS) PCR assay

**Figure 2:**
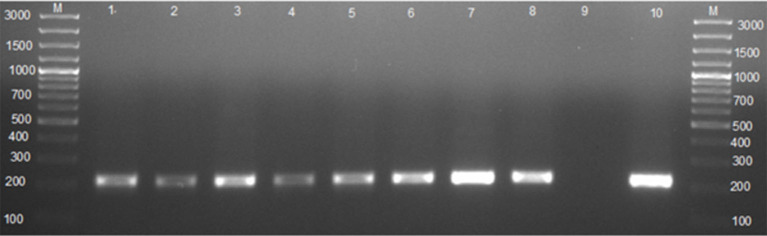
Agarose gel electrophoresis of the 16S−23S ribosomal DNA interspace region (ITS) PCR. Lane M: GeneRuler 100 bp (Thermo Fischer, Johannesburg, South Africa); lanes 1–3: *Brucella* spp. amplicon (214 bp) from whole blood; lanes 4–5: *Brucella* spp. amplicon (214 bp) from milk; lanes 6–8: *Brucella* spp. amplicon (214 bp) from vaginal swabs; lane 9: negative control containing sterile ultrapure water; lane 10: *B. abortus* 544.

Of the 118 cultured blood samples, 14 amplified a 214 bp specific amplicon of the genus *Brucella*. Of the 41 cultured milk samples, 4 from seropositive cows and 2 from seronegative cows amplified a 214 bp sequence of the genus *Brucella*, respectively (Table 3, Figure 2). In total, 6 milk samples were positive for 16S−23S ribosomal DNA interspacer region (ITS) PCR assay. Of the 51 vaginal swabs, 13 from seropositive cows and 2 from seronegative cows resulted in amplification of 214 bp sequence of the genus *Brucella*, respectively (Table 3, Figure 2). Of the 183 samples from seropositive cows, 31 were identified as of *Brucella* spp. whereas, out of the 27 samples from seronegative cows, 4 were identified as of *Brucella* spp. In total, out of the 210 samples that were inoculated on a modified CITA medium, 35 were ITS-PCR positive (Table 3, Figure 2). *Brucella* spp. were detected in 11.9% (14/118), 9.8% (6/41), and 29.4% (15/51) of the samples of whole blood, milk, and vaginal swabs, respectively. Altogether, *Brucella* spp. were detected in 16.7% (35/210) of seropositive herds of cattle farmed at the wildlife–livestock–human interface.

**Table 3:** Bacteriological, 16S−23S ribosomal DNA interspace region (ITS), AMOS, and Bruce-ladder PCR results of *Brucella* spp. isolated from seropositive herds of cattle farmed at the wildlife–livestock–human interface in Rwanda.

**Type of samples**	**Cultured**	**ITS**	**AMOS PCR assay**		**Bruce-ladder PCR**	
Whole blood	118	14	B.a	7	B.a	10
			B.a and B.m	3		
			B.m	1	B.m	4
			B.a and B.m	3		
Milk	41	6	B.a	4	B.a	5
			B.a and B.m	1		
Vaginal swabs	51	15	B.a	6	B.a	10
			B.a and B.m	4		
			B.a	2	RB51	2
			B.m	1	B.m	3
			B.a and B.m	2		

B.a: *Brucella abortus*; B.m: *Brucella melitensis*.

### Speciation of *Brucella* spp. using AMOS PCR assay

**Figure 3:**
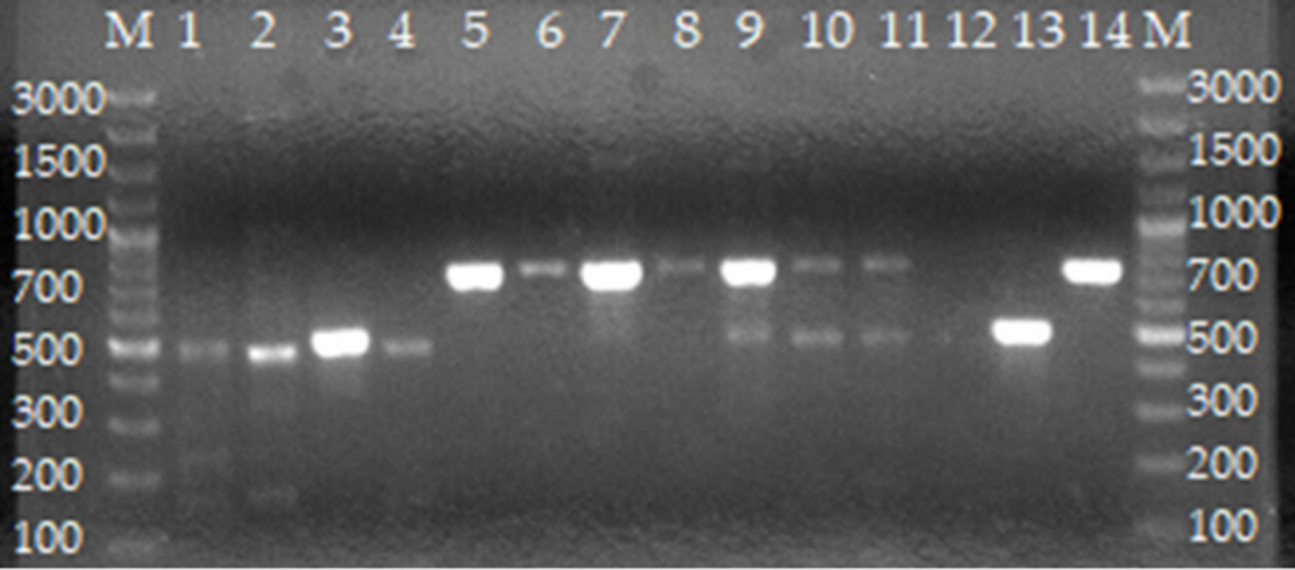
Agarose gel electrophoresis of AMOS PCR from cultures of isolates from cattle farmed at the wildlife–livestock–human interface. Lanes M: GeneRuler 100 bp (Thermo Fischer, Johannesburg, South Africa). Lanes 1–4: *B. abortus*; lanes 5–8: *B. melitensis*; lanes 9–11: mixed *B. melitensis* and *B. abortus*; lane 12: negative control containing sterile water; lane 13: *B. abortus* bv. 2 REF 544 strain; lane 14: *B. melitensis* bv. 1 16 M strain.

For whole blood, AMOS PCR assay identified mixed infections of *B. abortus* and *B. melitensis* (*n* = 6, simultaneous amplification of 731 and 496 bp), *B. abortus* (*n* = 7, amplification of 496 bp), and *B. melitensis* (*n* = 1, amplification of 731 bp) (Table 3, Figure 3). For milk samples, AMOS PCR assay identified one mixed infection of *B. melitensis* and *B. abortus* (*n* = 1) and *B. abortus* (*n* = 4) (Table 3, Figure 3). For vaginal swabs, AMOS PCR assay identified mixed infections of *B. melitensis* and *B. abortus* (*n* = 6), *B. abortus* (*n* = 8), and B. *melitensis* (*n* = 1) (Table 3, Figure 3).

### Distinction of terrestrial *Brucella* and vaccine strains using Bruce-ladder PCR assay

**Figure 4:**
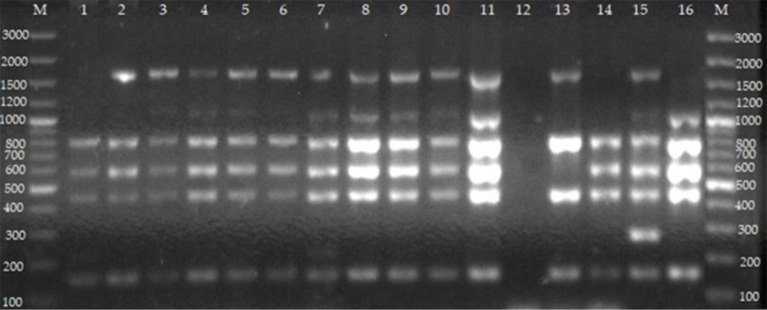
Agarose gel electrophoresis of Bruce-ladder PCR from cultures of isolates from cattle farmed at the wildlife–livestock–human interface. Lanes M: GeneRuler 100 bp (Thermo Fischer, Johannesburg, South Africa); lane 1: *B. abortus* RB51 from vaginal swabs; lane 2: *B. abortus* from whole blood; lanes 3–5: *B. melitensis* from whole blood; lane 6: *B. abortus* from milk; lanes 7–11: *B. melitensis* from vaginal swabs; lane 12: negative control containing sterile water, lane 13: *B. abortus* bv.1 S19; lane 14: *B. abortus* bv. 1 RB 51; lane 15: *B. suis* bv. 1 ZW 45; lane 16: *B. melitensis* bv. 1 16 M strain. Five PCR products (152, 450, 587, 794, and 1,682 bp) are expected for *B. abortus* (deletion of 25,061 bp in BMEII 0826–BMEII 0850). Five PCR products including 152, 450, 587, 794, and 2,524 bp are observed in *B. abortus* RB51 (insertion in BMEI 0998). Four PCR products including 152, 450, 794, and 1,682 bp are for *B. abortus* S19 (deletion of 702 bp in BMEII 0427–BMEII 0428). Six PCR products including 152, 450, 587, 794, 1,071, and 1,682 bp are expected in *B. melitensis* (insertion in BMEII 0843). Six PCR products including 152, 218, 450, 587, 794, and 1,682 bp are for *B. melitensis* rev 1 (point mutation in the BMEI 0752). Five PCR products including 152, 450, 587, 794, and 1,071 bp are for *B. ovis* (deletion of 1,507 bp in BMEI 0993–BMEI 1012). Six PCR products including 152, 272, 450, 587, 794, and 1,682 bp for *B. suis*. Five PCR products including 272, 450, 587, 1,071, and 1,682 bp are observed in *B. canis* (deletion of 976 bp in BME1435).

For whole blood, the Bruce-ladder PCR assay identified *B. abortus* (*n* = 10) and *B. melitensis* (*n* = 4) (Table 3, Figure 4). For individual milk samples, Bruce-ladder identified *B. abortus* (*n* = 5) (Table 3, Figure 4). For vaginal swabs, Bruce-ladder identified *B. abortus* (*n* = 10), *B. abortus* RB51 (*n* = 2), *B. melitensis* (*n* = 3) (Table 3, Figure 4).

The isolation of *Brucella* spp. was significantly associated with district with Nyagatare having more isolates [36.6%, (15/41)] than Nyabihu [36.4%, (4/11)], Kayonza [24.5%, (13/53)], Gatsibo [7.4%, (3/27)], and Musanze [0.0%, (0/9)] (*p* = 0.01).

## Discussion

*Brucella* spp. fall under category A pathogens and cause serious diseases in a wide range of animals and humans (1). Bovine brucellosis negatively affects national economies and public health worldwide (1, 36, 37). Seroprevalence studies showed that bovine brucellosis is prevalent in Rwanda (21, 22). However, serology does not provide a complete diagnosis and has drawbacks related to sensitivity and specificity (37, 38). Furthermore, *Brucella* spp. that are involved in the *Brucella* infections remain unknown in Rwanda. This study isolated *Brucella* spp. from blood, milk, and vaginal swabs of dairy cattle in Rwanda. The identified *Brucella* spp. included individual and mixed infections of *B. melitensis* and *B. abortus*. Two *B. abortus* RB51 were isolated from vaginal swabs using Bruce-ladder PCR assay.

The frequency of molecular detection of *Brucella* spp. (11.9%) from cultures of whole blood of cattle was higher than the finding (5.8%) of Caine et al. (39) and other previous studies which did not detect *Brucella* spp. from cultures of blood (40, 41). The present finding indicated that whole blood may be a good sample for isolation of *Brucella* spp. if processed immediately after collection (39, 42). The frequency of isolation of *Brucella* spp. in 12.2% of milk samples in this study is higher than the 6.5% recovered from raw milk informally marketed on streets in Uganda (43). The presence of *Brucella* spp. in milk is worrying since 21.7% of cattle keepers owning these seropositive cows reported drinking raw milk (21) which might be reflected in the human brucellosis cases detected in Rwanda (23, 24).

The isolation of *Brucella* spp. from seropositive and seronegative cows is consistent with earlier studies which also revealed the presence of *Brucella* spp. from seropositive and seronegative cows in Bangladesh (44), and China (45). The detection of *Brucella* spp. in seronegative animals may be because *Brucella* antibodies decrease in seronegative cows and chronically diseased cows while the organism remains intracellular in different tissues (44). The detection of *Brucella* spp. in seronegative cattle indicated that serological tests such as i-ELISA with cut-off points determined in Europe with no or low prevalence of brucellosis must be validated for Rwandan cattle. The isolation of *Brucella* spp. from the milk of seronegative cows is a problem of concern since serology is the only diagnostic method of brucellosis in Rwanda, and seronegative dairy cows continue shedding the pathogen in milk which is a valuable commodity, sometimes consumed unpasteurized and sold at the non-regulated market (21, 46). Furthermore, the traditional homemade cream milk known as “Ikivuguto” in the local language is frequently made of raw milk by several Rwandan families (47). Therefore, there is a need to investigate the presence of *Brucella* spp. in the homemade cream milk “ikivuguto” and to generate an awareness of this risk in Rwanda.

The detection of *Brucella* spp. in 29.4% of vaginal swabs was higher than the 12.6% previously reported in Pakistan (48), 1.5% in Mongolia (49), and 1.1% in Nigeria (50). This difference may be associated with the origin of samples and in this study, samples were collected from seropositive cows farmed in high-risk zones (21). In addition, the amount of *Brucella* isolation may also depend on the storage conditions and culture medium used (27, 51). The isolation of *Brucella* spp. from vaginal swabs confirms that *Brucella* organisms have the tropism for the reproductive organs of mature animals and massively multiply in the presence of reproductive hormones and erythritol (36, 52). Therefore, this finding support that vaginal swabs may be a good specimen for rapid molecular detection of brucellosis in animals (28).

*Brucella* spp. were more isolated from Nyagatare district compared to other districts. This difference may be due to the number of vaginal swabs (33/51) for the Nyagatare district compared to 5/50, 0/84, 7/13, and 6/12 for Gatsibo, Kayonza, Musanze, and Nyabihu districts, respectively. *Brucella* spp. colonize reproductive organs and were highly present and viable in vaginal swabs, which contained transport and storage medium (52). It was not surprising to detect *B. abortus* vaccine strain RB51 which is the vaccine used in the vaccination of cattle in high-risk zones in Rwanda. The identification of vaccine strains RB51 from cattle farmed at the interface in Rwanda indicates that RB51 is not safe for cattle and causes brucellosis in humans (53, 54) and that vaccinating pregnant animals should be done with caution.

It is of diagnostic importance that the 16S−23S ribosomal DNA interspace region (ITS) PCR detected *Brucella* DNA from seropositive cows as well as seronegative cows. The ITS PCR was able to detect as little as 3.8 fg of *B. canis* DNA mixed with 54 ng of template canine DNA extracted from vaginal swabs of non-infected bitches (28). The finding of this study confirms that ITS PCR can be used to detect *Brucella* spp. from vaginal swabs of animals that are seronegative, negative to blood culture, or blood PCR (28). However, there is a need to determine and validate the specificity and sensitivity of the ITS PCR in Rwanda since closely related *Brucella* pathogens that were not analyzed by Keid et al. (28) might be locally present and could generate false positives.

The recovery of *B. abortus* in the present study is consistent with earlier studies in the region (43, 55). This finding confirms that *B. abortus* is the main causal agent of brucellosis in dairy cattle. Although *B. melitensis* commonly cause the disease in goats, it was isolated in dairy cattle in the present study which could be due to the practice of co-rearing of animals (21). Hence there is a need to strengthen brucellosis control in cattle and avoid interspecies farming in Rwanda. AMOS-PCR detected a mixed infection of *B. abortus* and *B. melitensis* in the blood, milk, and vaginal swabs of cattle. Mixed infections of *B. abortus* and *B. melitensis* have been recently reported in aborted tissues of goats in Rwanda (26) and in herds where cattle graze together with small ruminants in South Africa and Kenya (56, 57). Keeping different animal species such as cattle and small ruminants on the same farm represents a risk of transmission of brucellosis to other animal species including humans. The purification of these cultures is recommended for future studies to isolate separately *B. abortus* and *B. melitensis* which primarily cause brucellosis in cattle and humans, respectively (36).

This is a problem of concern because diseased animals reduce production and *Brucella* spp. are present in the blood, milk, and vaginal secretions. This represents a great risk of contamination to handlers of live animals, carcasses, and consumers of raw milk and milk products.

## Conclusion

This study identified mixed and single infections caused by *B. abortus* and *B. melitensis* from whole blood, vaginal swabs, and milk indicating the great risk of transmission to handlers of live cattle, carcasses, and consumers of unpasteurized milk and milk products. We, therefore, advise the revision of the vaccination program to include protection against *B. melitensis* in livestock. The study also isolated *B. abortus* RB51, a vaccine strain, in seropositive cattle. Education about the epidemiology of brucellosis and other zoonotic diseases is of paramount importance to all stakeholders in the animal sector and consumers of animal products.

## Data availability statement

The original contributions presented in the study are included in the article/Supplementary material, further inquiries can be directed to the corresponding author.

## Ethics statement

The animal study was reviewed and approved by College of Agriculture, Animal Sciences and Veterinary Medicine, University of Rwanda (Ref: 026/DRIPGS/2017), Institutional Review Board of the College of Medicine and Health Sciences, University of Rwanda (No. 006/CMHS IRB/2018). Ethical clearance was also obtained from Animal Ethics Committee of the Faculty of Veterinary science, University of Pretoria, South Africa (V004/2018). Written informed consent was obtained from the owners for the participation of their animals in this study.

## Author contributions

JN and HH: conceptualization and methodology. JN: formal analysis and writing—original draft preparation. JN, EU, VM, AI, RG, and LU: investigation and data collection. JN, FK, EM, and HH: writing—review and editing. HH and FK: supervision. HH: project administration, resources, and funding acquisition. All authors have read and approved the manuscript.

## Funding

This study was supported by the Belgian Directorate-General for Development Cooperation, through its Framework Agreement with the Institute of Tropical Medicine (FA DGD-ITM 2017–2021). The funding body did not play a role in the design, analysis, and reporting of the study.

## Conflict of interest

The authors declare that the research was conducted in the absence of any commercial or financial relationships that could be construed as a potential conflict of interest.

## Publisher's note

All claims expressed in this article are solely those of the authors and do not necessarily represent those of their affiliated organizations, or those of the publisher, the editors and the reviewers. Any product that may be evaluated in this article, or claim that may be made by its manufacturer, is not guaranteed or endorsed by the publisher.
